# The Burden of Obesity in the Rural Adult Population of America

**DOI:** 10.7759/cureus.15770

**Published:** 2021-06-20

**Authors:** Okelue E Okobi, Olamide O Ajayi, Tobechukwu J Okobi, Ifeoma C Anaya, Oyinlola O Fasehun, Chiamaka S Diala, Endurance O Evbayekha, Abimbola O Ajibowo, Iyanu V Olateju, Joanna J Ekabua, Mireille B Nkongho, Ijeoma O Amanze, Anthonette Taiwo, Ovie Okorare, Ugochi S Ojinnaka, Omosefe E Ogbeifun, Nnenna Chukwuma, Emmanuel J Nebuwa, Janet A Omole, Iboro O Udoete, Rita K Okobi

**Affiliations:** 1 Family Medicine, Lakeside Medical Center, Belle Glade, USA; 2 Internal Medicine, Obafemi Awolowo College of Health Sciences, Olabisi Onabanjo University, Sagamu, NGA; 3 Internal Medicine, BronxCare Hospital, Bronx, USA; 4 Pathology and Laboratory Medicine, Faculty of Medicine, Ahmadu Bello University, Zaria, NGA; 5 Internal Medicine, University College Hospital, Ibadan, NGA; 6 Isolation/Internal Medicine, Stella Obasanjo Isolation Center, Benin City, NGA; 7 Internal Medicine, Lugansk Medical University, Lugansk, UKR; 8 Internal Medicine, Washington Adventist University, Takoma Park, USA; 9 Infectious Disease, University of Louisville, Louisville, USA; 10 Hematology, University of Virginia, Charlottesville, USA; 11 Psychiatry, Saint James School of Medicine, Saint Vincent, VCT; 12 Internal Medicine, Molly Specialist Hospital, Ibadan, NGA; 13 NA, USA; 14 Internal Medicine, Delta State University, Abraka, NGA; 15 Internal Medicine, Lankenau Medical Center, Wynnewood, USA; 16 Public Health, University of West Florida, Pensacola, USA; 17 Internal Medicine, Advocate Christ Medical Center, Oak Lawn, USA; 18 Internal Medicine, Rockville Ambulatory Surgery Center, Silver Spring, USA; 19 Internal Medicine, California Institute of Behavioral Neurosciences & Psychology, Fairfield, USA; 20 Public Health, Central Michigan University, Mount Pleasant, USA; 21 Research and Development, University of Maryland, Laurel, USA

**Keywords:** obesity, causes, associations, rural america, adult, factors, complications, prevalence

## Abstract

There is an epidemic of obesity in adults in rural America. It is estimated that about 19% of the population resides in rural areas, which encompasses 97% of America's total landmass. Although rural America makes up a fraction of America's total population, it has been estimated that the prevalence of obesity is approximately 6.2 times higher than in urban America. This illustrates an apparent disparity that exists between the rural population and urban populations that needs to be examined.

The prevalence of obesity, especially in rural America, is a growing concern in the medical community in recent years. Obesity has been identified as a significant risk factor for cardiovascular disease, cancer, and type 2 diabetes mellitus, which are leading causes of morbidity and mortality in the US. To better understand the disparity in the prevalence of adult obesity between rural and urban America, researchers have identified risk factors that are associated with the high incidence and prevalence of obesity in the rural American adult population. Low income and lack of physical activity have been identified as factors that predispose rural Americans to increased risk of obesity, arguing that low-income Americans may not have access to the resources available to assist them in weight reduction. With rural Americans being at an income disadvantage, it creates a risk for obesity, which further predisposes them to chronic diseases such as hypertension, obstructive sleep apnea (OSA), diabetes, and coronary artery disease.

As obesity continues to rise among the American population, the burden on the rural population is incredibly evident. Despite ongoing efforts by the US government and strategies implemented by the Common Community Measures for Obesity Prevention, there is still much to be done to tackle the epidemic. With an existing strategy in place, such as the 12 Common Community Measures for Obesity Prevention (COCOMO) strategies to fight obesity with physical activity, Americans are a step closer to conquering this epidemic. However, until other disparities such as income are addressed, rural Americans may continue to be severely impacted by the rising incidence of obesity and subsequent higher mortality rates from associated diseases.

## Introduction and background

Obesity has emerged as a global health crisis as various activities of daily living are beginning to favor a more sedentary lifestyle. There has been a massive increase in the prevalence of obesity across all age groups worldwide. In the last decade, the prevalence of adult obesity increased remarkably from 30.5% in 2000 to 40.4% in 2018 [1]. It is estimated that the severity of obesity in older adults will approach 82.3 million by 2040 [2]. In 2020, the Centers for Disease Control and Prevention (CDC) reported a greater than 40% prevalence of obesity in the U.S. Its prevalence remained significantly higher among adults residing in rural counties (34.2%) compared to those living in urban counties (28.7%) with over one-third of the American population classified as obese [3]. This is concerning considering the role of obesity as a major risk factor for several diseases, including cardiovascular diseases, cancers, type 2 diabetes mellitus, and metabolic disorders [4]. Obesity has also been linked to an early decline in mental, physical health, and cognitive function [5]. The prevalence of obesity is higher in rural areas than in urban areas [3]. In older adults living in rural areas, there is an increased risk of functional decline, threatening their ability to live independently [6]. Obesity is defined as a body mass index (BMI) of 30 kg/m^2^ or greater [7]. The BMI defines and sub-classifies obesity based on the individual's weight in kilograms divided by the square of the height in meters. Several factors have been shown to account for excess body fat, including genetics, sedentary lifestyle, and diet. These factors, either individually or together, ultimately lead to a caloric surplus converted and stored as fat in the adipocytes, tissues, and organs. Our objectives are to identify the burdens of obesity in the adult population in rural America and its various associations.

Methodology

Our search strategy and study selection are based on a defined set of inclusion and exclusion criteria. The search was conducted in PubMed, Google Scholar, and Cochrane Library. We included articles from the last five years (2016 to 2021) and considered randomized controlled trials, observation studies, reviews, and systemic reviews, as well as meta-analyses. Keywords for the search included obesity, causes, associations, rural America, adult, factors, complications, prevalence. We customized the searches according to the layout of each search engine and made every possible combination of the search keywords to generate the included articles.

Our keyword combinations and search results generated 80 articles from the various repositories. We screened the results by title to 64 matches. We read the abstracts considering our objectives, inclusion, and exclusion criteria, then further narrowed them down to 43 studies in full text and analyzed 38 articles that directly documented the various facets of obesity in rural America. Upon reading the articles, the information was divided into groups, making associations, regions, race, physical activities, income, perception, healthcare access, and policy and mortality into consideration.

## Review

Eligibility criteria

Articles reviewed included case-control studies reviews, systemic reviews, cohort studies, and randomized clinical controlled trials that focus on obesity in adult Americans living in rural communities. We defined rural America using the US Office of Management and Budget definition [8]. Articles that did not focus on rural America and were outside the selected date range (2016 to 2021) were excluded from this review.

Discussion

With approximately 19% of the population living in the rural areas of America according to the 2010 census [8], over 60 million people are constantly barraged by health challenges. Among the health challenges of rural settlers, obesity has been reported to be on the rise compared with the urban regions of the US. Several pertinent aspects of obesity have been studied and documented.

Over one-third of Americans, 65 years and over, are affected by obesity, with about one in seven adults having a body mass index (BMI) of 35 kg/m^2^ or greater. The prevalence of obesity (BMI > 30) in older adults has increased by 4.4% from 2003 to 2012. This is an exceptional obesity growth rate compared to other age groups. Findings from different studies suggest there is a significant relationship between urban-rural status and obesity. Obesity rates are higher in rural areas compared to urban areas. The key contributing factors to obesity are unhealthy diet habits, little to no physical activity, demographic, socioeconomic, low educational attainment, biological, genetic, increased number of fast-food outlets, and decreased availability of healthy food choices [9].

Food and income distribution

Many people who reside in rural communities lack facilities and resources that can enable them to adopt positive dietary practices due to their low socioeconomic status [10]. This dietary component of obesity was reflected in a study in an African American community in Mississippi, in which 70 focus group members participated in interviews about dietary practices [9]. The study suggested that unhealthy nutritional practices and limited access to healthy food options are significant factors that promote obesity, particularly in rural communities.

The neighborhood food environment also shapes the dietary patterns of the people inhabiting a territory. Valdez et al. [11], in December 2016, focused on the food environment as the obesity rate in the Latino community in the US was on the rise. This study observed an association between lack of physical access to affordable produce in areas where grocery stores and supermarkets are limited and poor dietary intake with obesity. This association was high in rural low-resource communities with a high population of Latino residents. They studied 79 residents with a mean age of 41.6 years and reported that dollar and discount stores in this agricultural area provided access to produce. However, produce at retail stores was less affordable than produce at non-retail stores such as fruits and vegetables stand. This shows that lack of access to healthy produce by food retailers and affordability of the food produce within those retail outlets influence dietary intake by limiting residents' consumption of fruits and vegetables. This relationship is linked to diet-related behaviors associated with obesity and preventable chronic diseases [11].

In a cross-sectional analysis carried out on 50,884 women who enrolled in the sister study between 2003 and 2009, the study showed that women who lived in areas of the highest “tertile of greenness” had a reduced risk of obesity relative to those who lived in the lowest “tertile of greenness.” This bridged the gap between studies that showed that those who live in greener areas are more physically active and have lower rates of obesity. Beneficial associations between greenness, obesity and physical activity were observed in urban and rural areas, and regionally, stronger associations were observed in the western census region in the US [12].

The role of the available food environment has also been studied in rural demographic populations. A study examined the association between food environments (characterized as food swamps, food deserts, or others) and obesity rates among US adults. Source data were a combination of food outlet, sociodemographic, and obesity data from the American Community Survey (ACS), the US Department of Agriculture (USFDA), Food Environment Atlas, and a commercial food street reference dataset. Three food stamp measures were used in this study: (1) Traditional Retail Food Environment Index (RFEI); (2) Expanded RFEI #1; and (3) Expanded RFEI #2. Even after controlling for food desert effects, the authors found that food swamps have a positive, statistically significant effect on adult obesity rates. All three food swamp measures indicated the same positive association but reflected different magnitudes of the food swamp effect on rates of adult obesity (p-values ranged from 0.00 to 0.16). Our adjustment for reverse causality, using an IV approach, revealed a stronger effect of food swamps than would have been obtained by naïve ordinary least squares (OLS) estimates. The food swamp effect was stronger in counties with greater income inequality (p < 0.05) and where residents are less mobile (p < 0.01). This study examined mainly urban and suburban areas, but there were inclusions of rural sections of the US population. The study can potentially help address obesity prevalence rates in rural areas by combining local government policies such as zoning laws that restrict access to unhealthy food sources while enhancing the entry of healthy food sources to localities [13].

Lenardson, Hansen, and Hartley noted factors that influence the health of rural people to include climate culture references, transportation access, and remoteness [14]. They observed that many rural people are likely to have a low income, which significantly affects the quality of their diet. They attributed this low income to their low educational attainment in rural regions, contributing to their lifestyle and nutrition choices. They recommended that health policies and programs should focus on the specific needs of the communities.

Pitts et al. [15], in three rural counties in North Carolina, assessed the effect of Farmers' Market Initiatives among the residents as an obesity prevention strategy targeting the underserved population. Parameters measured included awareness, use, hindrances, and facilitators. Over one year, the initiatives had varying effects across the counties. There were increases, decreases, and static changes. The variation can be attributed to accessibility, availability of alternatives, odd market hours, and perceived affordability. However, the study was limited by a short follow-up time of one year, which was insufficient to measure appreciable changes in the parameters [15].

Income inequality is another contributory factor. Fitch et al. noted the dissimilarity in obesity across counties in the US. This was attributed to the inability of low-income families to afford sporting activities such as gym memberships and sporting gears. Also, the lower consumption of fruits and vegetables alongside the easy access to fast foods has resulted in a prevalence of obesity among rural adults to be 39.6% compared to urban figures 33.4% [16].

These income disparity concerns were also documented by a study done by Tomayko et al. [17]. The tools used for analysis in the study were the United States Department of Agriculture (USDA) and the dietary screener questionnaire. Their findings were that families living in rural communities found their proximity to essential food groups a concern, making fewer trips than their counterparts living in urban areas. Also, income disparities between them and their urban counterparts showed that higher-income individuals made more trips to the grocery store and had varieties to choose from, leading to more income being spent on food. The study concluded that exposure to other food groups and proper nutritional education would improve obesity outcomes in both children and adults [17].

Healthcare policy and access to care

The study by Harris et al. [18] reported that health outcomes for rural residents were influenced by a combination of low-performing health departments and environmental and individual characteristics. Factors such as poverty, high-risk health behavior like smoking, poor access to healthcare, poor health quality, and weak public health policy affected health outcomes in these rural demographics. The study also noted that rural local health departments had a fewer staff, less specialization, limited access to technology, electronically available information such as public health evidence and training opportunities. It highlighted harmonization, partnership, and consolidation of rural jurisdiction as potential actions to address these challenges in rural local health departments. Furthermore, the authors recommended that policymakers and public health care practitioners tackle risky health behaviors and poverty to improve rural health care [18]. Some authors also attributed proximity and access to a healthcare facility as risk factors for obesity. In a study [19], they found that rural access to qualified clinicians and dieticians was limited, worsened by transportation and financial barriers. They studied 138 patients with BMI > 30 and ages 18 years and older were therefore carried out. Eight bi-weekly sessions were provided via telemedicine video conferencing for these patients at the rural primary care clinics. The result of the study emphasized the importance of the use of innovative telemedicine interventions to bridge the gap and alleviate the barriers to accessing evidence-based services to decrease obesity rates in rural communities in the US [19].

Access to health resources was also addressed in an article by Rural Health Information Hub. They noted that people in rural areas experience higher rates of obesity than the urban areas. However, they stated that most rural communities do not have the resources required to address obesity health concerns. Their submission suggests that residents in rural areas must travel long distances to access health facilities, exercise facilities, and healthy foods. Health facilities in rural areas are less likely to have dietitians, nutritionists, or weight management experts [19]. They concluded that health facilities in rural areas could develop programs and services that help the residents learn about the health risk of overweight and obesity to address the problem.

Obesity perception

How residents in this demographic region perceive the burden of obesity was another concern. A study comprising 538 individuals from 12 rural counties in West Virginia found a wide gap in reported lifestyle behavior among participants and their overweight and obesity Status. 71% of the undiagnosed high-risk individuals indicated that they read food labels when making food choices. Yet, nearly half of the participants were obese according to their self-reported BMI Rankings. The study also found a lack of perceived personal risk among those with a family history of diabetes and hypertension, indicating that participants did not apply the knowledge to themselves [20].

Physical activity

The rural residents were less physically active than their urban counterparts. They were disproportionately affected by chronic diseases and conditions associated with calorie imbalance, which included obesity. Researchers have found reduced documentation of the physical activity levels in rural areas compared to urban areas. They suggested a need for more theoretically supported, methodologically rigorous, and empirically tested "rural strategies" for intervention on physical activity in rural America [21]. In a pilot study, Batsis et al. conducted lifestyle intervention among rural obese adults, behavioral changes, and increased physical activity using wearable activity devices like Fitbit. It was noted from the interviews that there was an increased enthusiasm in the use of activity feedback, self-monitoring, and motivation to lose weight. Although usability and satisfaction were observed, there was decreased exercise confidence, and patient activation was no different pre/post-pilot study. The results suggest that the use of electronic devices and their information may not lead to behavioral activation to lose weight alone. It is essential to include social interaction and group engagement among older adults in rural America to motivate and inspire behavioral change in this high-risk population [4].

Race

According to a review done by Florez et al. [22], obesity was predominant amongst Latino and African American females in rural communities. They acknowledged that a limitation in their study is a probable underrepresentation of men and women of other races, which might affect the reliability of results and outcomes generated from such studies [22]. Studies have also proven the postulated disparities of obesity among ethnic groups. To put this concept in perspective, the prevalence of obesity is reported to be less in White women (31%), compared to Latina women (41%) and African American women 51% [23,24]. Many other researchers have contributed to the likely etiology of these disparities; in terms of genetics, lifestyle, access to education, socioeconomic status, etc.; research is still ongoing in this area [25]. Torres-Anguilar et al. conducted a study on immigrant Latinos in non-metropolitan rural communities. They concluded that environmental and lifestyle factors play a significant role in dietary behaviors or choices [25].

Social factors

Previous studies highlighted the role of social factors on eating behavior, obesity, and smoking cessation among residents in rural America [26]. Seguin and his group researched how social relationships influence the adoption and maintenance of health-related behaviors among midlife and older adults in rural Montana. The study found that family interactions and peer influences dictated many health-related decisions people made inside and outside the home. Positive role modeling and encouragement from family members also emerged as important facilitators of physical activity and smoking cessation. This supportive team mentality provides the motivation and accountability needed to stick with the initiated lifestyle changes.

Prevention

Various interventional models to decrease obesity were noted in the studies reviewed. The elderly people, >65 years and older, are the fastest-growing population at risk of obesity in rural America [27]. Batsis et al. [28] described the effects and challenges facing implementing the Medicare Obesity Benefit (MOB) Regulatory Coverage introduced by the Centers for Medicare and Medicaid Services (CMS) in November 2011. The program was meant to promote quality obesity care delivery to medicare beneficiaries by Primary Care Physicians (PCPs). MOB entails providing up to 22 visits of intensive behavioral therapy (IBT) targeted at weight loss over a 12-month window, and recipients were expected to achieve a weight loss of 3 kg in six months to maintain eligibility. The challenges with its implementation within a rural primary care setting included the scarcity of workforce and specialized services, long-distance/transportation barriers, and a poor reimbursement mechanism currently at $27 per session. Recommendations to increase the utilization of MOB between PCPs and patients included telemedicine as a substitute for face-to-face visits, incorporating other providers like the peer-health coaches, dietitians, and raising reimbursements or switch to value-based care [28].

A study of African American women from rural areas of Alabama and Mississippi found that trained lay staff and volunteers were able to effectively deliver a translation of an evidence-based behavioral weight loss intervention to African American women who reside in rural Southern communities and thus, the use of trained lay staff is a promising approach for the reduction of obesity in rural southern communities [29]. Another strategy to mitigate this problem in these rural demographics was reviewed by Batsis et al. [30]. They thought that this fast-growing adult population of rural America is seldom unaware of behavioral modification and lifestyle management strategies to mitigate this problem, for example, dietary counseling and exercise. They reported that a 26-week mobile health obesity wellness intervention program via videoconferencing showed that using technology (a self-monitoring mHealth device) to combat obesity in rural areas is promising and could enhance healthcare promotion in remote rural communities. The program included intensive behavioral therapy, calorie restriction, and increased physical activity via motivational interviewing, twice-monthly group exercise, and dietary counseling. The program showed that elimination of physical presence, distance, and frequent traveling to receive care were factors that promoted behavioral change and weight loss. They recommended that telehealth delivery methods should be used to combat obesity in rural communities. [30]. Numerous interventions, strategies, and health promotion programs have been implemented to curtail the obesity epidemic in rural America. These interventions include strengthening food networks between suppliers and retailers to provide fresh, healthy foods to the community. Other strategies implemented to prevent obesity in rural areas include increasing physical activities by the use of bicycle paths and outdoor recreational facilities. Farley et al. [31] researched to evaluate the ability of a multichannel mass media campaign to reduce sugar-sweetened beverages (SSB) consumption in rural northeast Tennessee, southwest Virginia and southeast Kentucky, which had high rates of SSB consumption. The evaluation involved subjective and objective measures before and after the intervention area and a matched comparison area. The survey results showed significant differences in participant beliefs about SSBs from before to after the campaign; the study campaign succeeded in increasing awareness among participants about the role of SSB in weight gain, heart disease, dental caries, and obesity.

Mortality

Obesity is a leading cause of death in the US. It severely impacts people's quality of life, increases vulnerability to heart diseases, stroke, kidney failure, and blindness. CDC [32] reported that obesity has been linked to a higher rate of mortality in rural areas of the United States. Studies have shown that residents of Appalachian counties have an increased prevalence and mortality from obesity and obesity-related chronic conditions than those living outside the region [33]. West Virginia accounts for the highest prevalence of obesity in the Appalachian region and the US. The aging population, physical inactivity, obesity, geography, lack of access to quality care, and the Appalachian culture of distrust of the healthcare system contribute to obesity in West Virginia.

Limitations

 Articles, clinical trials, opinions reviewed were regional and may not be generalizable to every rural community. Also, study bias, sample sizes, and limited available research articles were noted during this review. Some areas of obesity were not covered in this review due to limited resources.

## Conclusions

The burden of obesity in rural America requires diligent attention. This rising prevalence of this health challenge threatens the lives of over 60 million residents in this demographic region. Diet, lifestyle, access to healthcare, income, and education play a role in fueling this disease burden. Several other aspects of obesity in rural America are yet to be explored. Medical literature has demonstrated risk factors, for example, genetics, which was not included in this review. This leaves a call for a more rural-focused approach to the problems of obesity. We recommend the further rural-focused study to explore these and other pertinent aspects of obesity that may determine prevention, management approaches, prognosis, outcomes, and perhaps reduce mortality and curb this increasing health burden in our rural America.
